# Gastroduodenal Artery Pseudoaneurysm, A Rare Cause of Gastrointestinal Bleeding Complicating Pancreatitis

**DOI:** 10.14309/crj.0000000000001838

**Published:** 2025-10-02

**Authors:** Abraham Carboo, Mohammad Ramadan, Patrick Kwaah, Pia Dogbey

**Affiliations:** 1 Department of Medicine, Waterbury Hospital, Waterbury, CT; 2 Yale School of Medicine, New Haven, CT

**Keywords:** acute pancreatitis, pseudoaneurysm, gastrointestinal bleeding, coil embolization

## Abstract

Gastroduodenal artery pseudoaneurysm is an uncommon but life-threatening complication of acute pancreatitis (AP). We present a 58-year-old man with presumed mild AP who developed massive intra-abdominal hemorrhage due to a gastroduodenal artery pseudoaneurysm. The patient's initial imaging suggested a pancreatic pseudocyst, but an atypical presentation for AP prompted a computed tomography angiogram that identified an actively bleeding pseudoaneurysm. He underwent urgent coil embolization with successful hemorrhage control. This case highlights the importance of early recognition and multidisciplinary management of visceral artery pseudoaneurysms in pancreatitis to prevent potentially catastrophic outcomes.

## INTRODUCTION

Visceral artery pseudoaneurysms are a rare vascular complication of acute pancreatitis (AP), occurring in less than 10% of cases.^1,2^ Unlike true aneurysms, pseudoaneurysms do not consist of all 3 layers of the arterial wall and carry an exceptionally high risk of mortality from rupture and hemorrhage.^1,3,4^ Without a timely diagnosis and intervention, the mortality of a ruptured pseudoaneurysm is higher than that from other more common pancreatitis-related complications such as necrosis, abscesses, or pseudocysts and can approach over 50%.^5,6^ Gastroduodenal artery (GDA) pseudoaneurysms are one of the more frequently involved visceral artery pseudoaneurysms (24%) after splenic artery pseudoaneurysms (30%–50%).^7,8^ Early identification is challenging because initial symptoms may mimic uncomplicated pancreatitis. We present a case of massive hemorrhage from a GDA pseudoaneurysm complicating a presumed mild AP, highlighting the need for prompt recognition and multidisciplinary intervention.

## CASE REPORT

**Figure 1.**
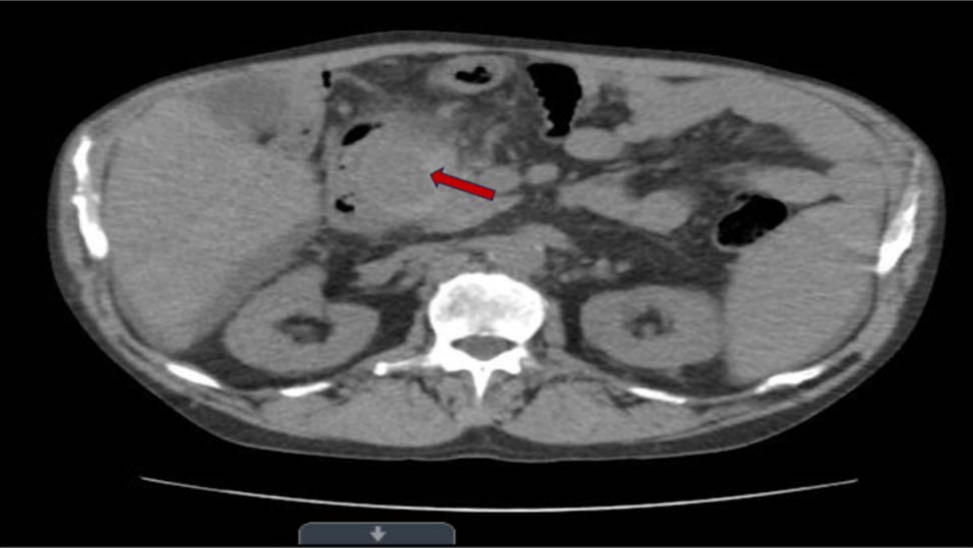
Axial computed tomography abdomen without intravenous contrast demonstrating peripancreatic inflammation around the pancreatic head. A presumed pancreatic pseudocyst is indicated (red arrow).

A 58-year-old man with a history of hypertension, alcohol-induced liver cirrhosis decompensated by ascites diagnosed 9 months before admission, and remote nephrolithiasis presented to the emergency department with 2 days of severe central abdominal pain radiating to the left flank, accompanied by nausea and vomiting. On admission, he was hemodynamically stable (Table 1). Physical examination revealed a soft abdomen with diffuse mild tenderness and no peritoneal signs. His initial laboratory tests showed hemoglobin of 12.4 g/dL, an amylase of 146 IU/L (reference 30–110), and a of lipase of 441 IU/L (reference 23–300) (BISAP score-0), concerning for mild AP. Abdominal ultrasonography demonstrated no gallstones but did reveal complex cystic lesions in the pancreas. An initial noncontrast computed tomography (CT) scan of the abdomen and pelvis showed 2 low-density lesions in the pancreatic head suggestive of pseudocysts and minimal perihepatic and peri splenic free fluid (Figure 1). The patient was initially admitted for management of presumed mild AP, with fluid resuscitation, analgesia, and bowel rest while being monitored closely. Notably, the finding of pseudocysts during what was thought to be a first episode of AP was unusual, raising the possibility of unrecognized recurrent or chronic pancreatitis. Cysts were also noted to be present and enlarging on review of imaging from outside records 9 months before admission with no additional clinical information.

**Table 1.** Clinical course of patient

	Day of admission	25 hrs later	30 hrs later	At discharge
Pulse, bpm	84	89	107 H	74
BP, mm Hg	110/76	93/62	78/51 L	95/60
Hemoglobin, g/dL	13.6	10.3 L	6.1 L	8.6 L
WBC, 10^6^/uL	10.4	19.3 H	—	8.2
Platelets, 10^3^/uL	184	210	—	150
Serum amylase, U/L	146 H	—	—	—
Serum lipase, U/L	441 H	—	—	—

BP, blood pressure; H, high; L, low; WBC, white blood count.

**Figure 2.**
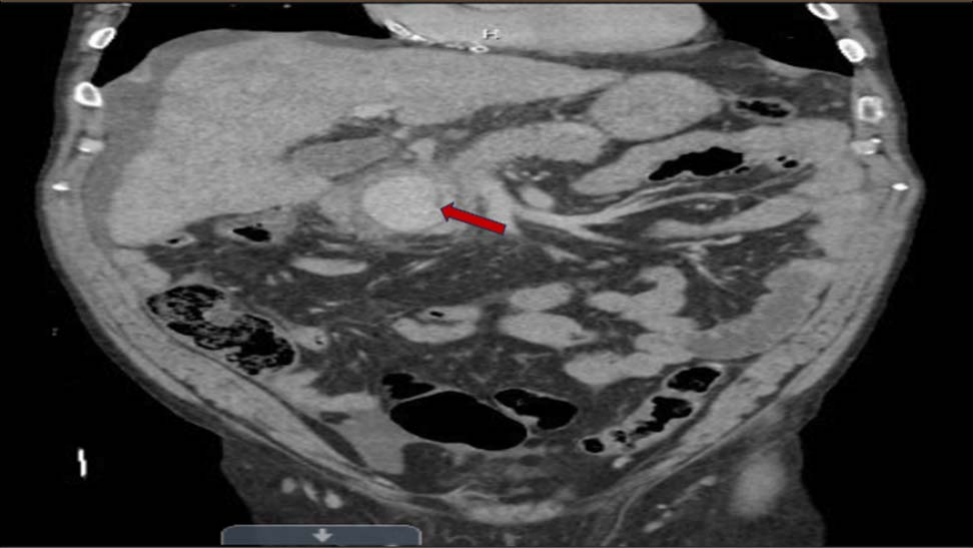
Coronal angiographic image during interventional radiology procedure showing a gastroduodenal artery pseudoaneurysm (red arrow).

**Figure 3.**
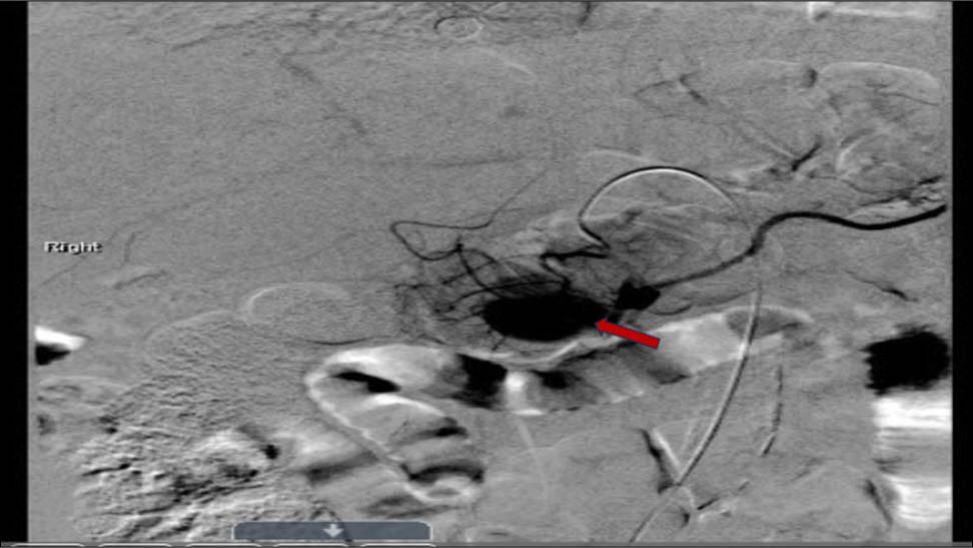
Coronal angiographic image before endovascular intervention. The gastroduodenal artery pseudoaneurysm is visualized before coil embolization (red arrow).

**Figure 4.**
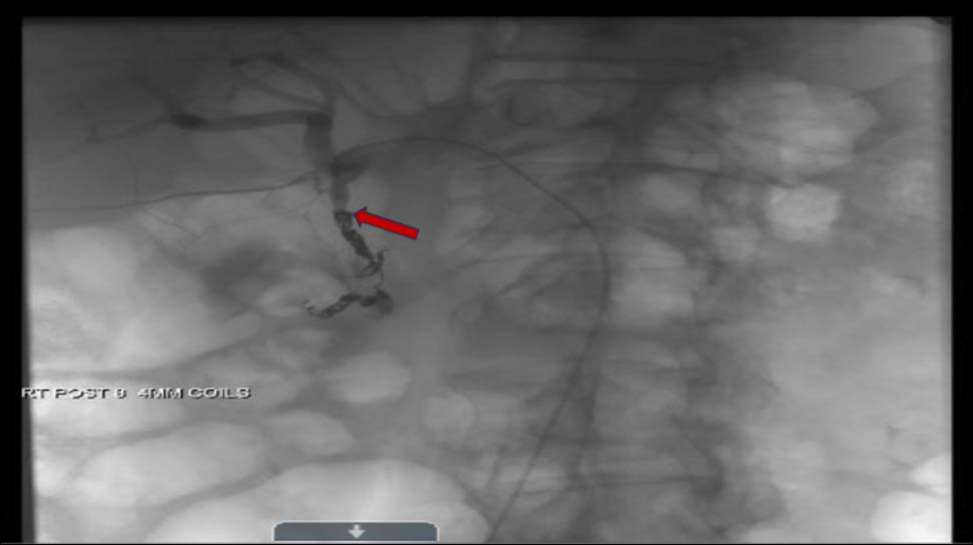
Coronal angiographic image after endovascular coiling, demonstrating successful embolization of the gastroduodenal artery pseudoaneurysm (red arrow).

This discrepancy in the clinical presentation of AP and unusual imaging findings for the duration of symptoms prompted further evaluation for alternative diagnoses, including mesenteric or portal vascular thrombosis, and an urgent CT angiogram was performed (Figure 2). The CT angiography revealed a 4.9 × 6.3-cm hematoma between the pancreatic head and proximal duodenum with active contrast extravasation from the GDA (Figure 2), indicating a bleeding pseudoaneurysm. Shortly after this finding, the patient became hypotensive (blood pressure 74/53 mm Hg), and his hemoglobin dropped acutely from an initial hemoglobin of 12.4 g/dL on admission to 7.2 g/dL, consistent with massive intra-abdominal hemorrhage (Table 1). He was transferred to the intensive care unit for resuscitation, and emergent consultation with general surgery and Interventional Radiology was obtained. Subsequent arteriography confirmed an approximately 5-cm pseudoaneurysm from the proximal to mid-GDA. The patient underwent urgent endovascular treatment with coil embolization of the pseudoaneurysm (Figures 3 and 4). The procedure successfully stopped the bleeding. The patient's condition stabilized, and he recovered without further complications. After hemotransfusion, he was discharged in stable condition with a hemoglobin of 8.6 g/dL, and arrangements were made for close outpatient follow-up.

## DISCUSSION

This case emphasizes the importance of vigilance for rare but life-threatening complications in suspected AP. Although AP generally has a low mortality rate (often well under 5% in mild cases),^9^ our patient's course was nearly fatal due to an atypical complication—a ruptured GDA pseudoaneurysm. Visceral artery pseudoaneurysms are estimated to occur in under 10% of pancreatitis cases,^1,2,10^ yet if one forms and ruptures, mortality can be exceedingly high (reported over 50% without treatment).^5,6^ Early recognition is challenging because the initial presentation can resemble routine pancreatitis or be clinically silent.^11^ In this case, the presence of pancreatic pseudocysts was an important clue for an unusual presentation, prompting imaging that led to the diagnosis. This reflects a key point: physicians should maintain a high index of suspicion for vascular complications like pseudoaneurysm when a patient with pancreatitis (acute or chronic) has atypical features or deteriorates unexpectedly.

Early cross-sectional imaging, particularly CT angiography, is crucial for the diagnosis. CT angiography is considered the gold standard for detecting visceral pseudoaneurysms due to its high sensitivity (95%–100%).^12^ Once a pseudoaneurysm is identified, prompt multidisciplinary intervention is required. Endovascular interventions by an interventional radiologist are often the first-line treatment, as they can rapidly control hemorrhage minimally invasively.^3,13,14^ In our patient, coil embolization of the GDA pseudoaneurysm successfully stopped the bleeding and likely averted surgical intervention. Endovascular therapy generally offers advantages in lower morbidity compared to open surgery. Surgical management such as vessel ligation may be necessary if embolization fails or if rebleeding occurs​.^15^ Close collaboration between gastroenterologists, radiologists, surgeons, and critical care teams is essential in managing such cases, given the potential for rapid hemodynamic deterioration and the need for intensive care and possible transfusion support.

Our case adds to the body of literature demonstrating that even a case of pancreatitis that appears mild at first can harbor a devastating vascular complication. It highlights the need for clinicians to be aware of pseudoaneurysm formation as a differential diagnosis when unusual clinical signs accompany pancreatitis, whether acute or chronic. With timely angiographic evaluation and intervention, patients with this complication can have favorable outcomes.

## DISCLOSURES

Author contributions: Conceptualization: A. Carboo, M. Ramadan, P. Kwaah, P. Dogbey. Data Curation: A. Carboo, P. Dogbey, Writing-original draft: A. Carboo, M. Ramadan, P. Kwaah, P. Dogbey. Writing-review and editing: A. Carboo, M. Ramadan, P. Kwaah, P. Dogbey. Supervision: P. Dogbey. P. Dogbey is the article guarantor.

Financial disclosure: None to report.

Previous presentation: The abstract of this case reported was presented at the Connecticut Chapter of the American College of Physicians (CT-ACP) scientific meeting on Friday; October 18, 2024; Plantsville, CT.

Informed consent was obtained for this case report.

## ABBREVIATIONS:

AP: acute pancreatitis
BISAP: BUN (Blood urea nitrogen), impaired mental status, SIRS (systemic inflammatory response syndrome), Age, Pleural effusion
BP: blood pressure
CT: computed tomography
GDA: gastroduodenal artery
H: high
IU/L: international units per litre
L: low
WBC: white blood count

## References

[1] Carr JA. Visceral pseudoaneurysms due to pancreatic pseudocysts: Rare but lethal complication of pancreatitis. J Vasc Surg. 2000;32:722–30. 11013036 10.1067/mva.2000.110055

[2] Dörffel T Wruck T Rückert RI Romaniuk P Dörffel Q Wermke W. Vascular complications in acute pancreatitis assessed by color duplex ultrasonography. Pancreas. 2000;21(2):126–33. 10975705 10.1097/00006676-200008000-00004

[3] Barge JU Lopera JE. Vascular complications of pancreatitis: Role of interventional therapy. Korean J Radiol. 2012;13(Suppl 1):S45–S55. 22563287 10.3348/kjr.2012.13.S1.S45 PMC3341460

[4] Sharma PK. Hemorrhage in acute pancreatitis: Should gastrointestinal bleeding be considered an organ failure? Pancreas. 2008;36:141–5. 18376304 10.1097/MPA.0b013e318158466e

[5] Lu M Weiss C Fishman EK Johnson PT Verde F. Review of visceral aneurysms and pseudoaneurysms. J Comput Assist Tomogr. 2015;39(1):1–6. 25319606 10.1097/RCT.0000000000000156

[6] Balachandra S Siriwardena AK. Systematic appraisal of the management of the major vascular complications of pancreatitis. Am J Surg. 2005;190(3):489–95. 16105542 10.1016/j.amjsurg.2005.03.009

[7] Maatman TK Heimberger MA Lewellen KA . Visceral artery pseudoaneurysm in necrotizing pancreatitis: Incidence and outcomes. Can J Surg. 2020;63(3):E272–E277. 32436687 10.1503/cjs.009519 PMC7829015

[8] Kalas MA Leon M Chavez LO Canalizo E Surani S. Vascular complications of pancreatitis. World J Clin Cases. 2022;10(22):7665–73. 36158481 10.12998/wjcc.v10.i22.7665 PMC9372863

[9] Ingraham NE King S Proper J . Morbidity and mortality trends of pancreatitis: An observational study. Surg Infect (Larchmt). 2021;22(10):121–1030. 34129395 10.1089/sur.2020.473 PMC8851213

[10] Lee Y Hsu C Chen K. Gastroduodenal artery aneurysm/pseudoaneurysm: A systematic review of reported cases. PeerJ. 2025;13:e19115. 40115272 10.7717/peerj.19115 PMC11925042

[11] Hoilat GJ Mathew G Ahmad H. Pancreatic Pseudoaneurysm. Treasure Island, FL: StatPearls Publishing LLC; 2023. 28613687

[12] Savastano S Feltrin GP Antonio T Miotto D Chiesura-Corona M Castellan L. Arterial complications of pancreatitis: Diagnostic and therapeutic role of radiology. Pancreas. 1993;8(6):687–92. 8255884 10.1097/00006676-199311000-00004

[13] Kukliński A Batycki K Matuszewski W Ostrach A Kupis Z Lęgowik T. Embolization of a large, symptomatic splenic artery pseudoaneurysm. Polish J Radiol. 2014;79:194–8. 10.12659/PJR.889974 PMC4089775 25009678

[14] Pang TCY Maher R Gananadha S Hugh TJ Samra JS. Peripancreatic pseudoaneurysms: A management-based classification system. Surg Endosc. 2014;28(7):2027–38. 24519028 10.1007/s00464-014-3434-9 PMC4065337

[15] Venturini M Marra P Colombo M . Endovascular repair of 40 visceral artery aneurysms and pseudoaneurysms with the viabahn stent-graft: Technical aspects, clinical outcome and mid-term patency. Cardiovasc Intervent Radiol. 2018;41(3):385–97. 29164308 10.1007/s00270-017-1844-5

